# Relevance of blood tumor markers in inpatients with significant involuntary weight loss and elevated levels of inflammation biomarkers

**DOI:** 10.1186/s12885-024-12201-0

**Published:** 2024-04-15

**Authors:** Morgane Gronnier, Kaies Hedhli, Chloé Sauzay, Valéry Salle, Pierre Duhaut, Jean Schmidt, Amandine Dernoncourt

**Affiliations:** 1 Department of Internal Medicine, Amiens-Picardie University Medical Center, Rue du Professeur Christian Cabrol, F-80054 Amiens, France; 2 Laboratory of Hematology, Center of Human Biology, Amiens-Picardie University Medical Center, F-80054 Amiens, France; 3 Laboratory of Biochemistry, Center of Human Biology, Amiens-Picardie University Medical Center, F-80054 Amiens, France; 4 RECIF, Amiens-Picardie University Medical Center, F-80000 Amiens, France

**Keywords:** Tumor biomarkers, Cancer, Inflammation, Weight loss, Diagnostic test, Computed tomography

## Abstract

**Purpose:**

To assess the diagnostic performance of a panel of standard tumor markers (TMs) in patients hospitalized with significant involuntary weight loss (IWL) and elevated levels of inflammation biomarkers, and a combination of the TM panel and the finding of the computed tomography (CT) scan.

**Methods:**

We conducted a retrospective study in the internal medicine department at Amiens-Picardie University Medical Center (Amiens, France) between January 1st, 2015, and November 1st, 2021. The inclusion criteria were age 18 or over, significant IWL (≥ 5 kg over 6 months), elevated inflammation biomarkers (e.g. C-reactive protein), and assay data on two or more standard TMs (carcinoembryonic antigen (CEA), carbohydrate antigen (CA) 19 − 9, CA 15 − 3, CA 125, neuron-specific enolase (NSE), alpha-fetoprotein (AFP), calcitonin, and prostate-specific antigen (PSA)). The result of each TM assay was interpreted qualitatively (as positive or negative), according to our central laboratory’s usual thresholds.

**Results:**

Cancer was diagnosed in 50 (37.0%) of the 135 patients included. Positivity for one or more TMs had a positive predictive value (PPV) of 0.55 [0.43–0.66], and a negative predictive value (NPV) of 0.84 [0.75–0.93] for cancer diagnosis. When combined with the presence of suspicious CT findings (e.g. a mass, enlarged lymph nodes and/or effusion), positivity for one or more TMs had a PPV of 0.92 [0.08–0.30]. In the absence of suspicious CT findings, a fully negative TM panel had an NPV of 0.96 [0.89-1.00].

**Conclusion:**

A negative TM panel argues against the presence of a cancer, especially in the absence of suspicious CT findings.

**Supplementary Information:**

The online version contains supplementary material available at 10.1186/s12885-024-12201-0.

## Introduction

Early diagnosis of cancer is essential for the initiation of effective treatments and the greatest possible benefit for survival [1]. The clinical signs of a progressing cancer depend on the affected organ, the histological type, and the stage at diagnosis [2]. Significant involuntary weight loss (IWL, defined as loss of at least 5% of the usual body weight over a period of 6 months or less, in the absence of a low-calorie diet or treatments producing weight loss) is a frequent, non-specific organ symptom often associated with an underlying cancer [2–4]. Indeed, between 25% and 30% of patients with IWL are diagnosed with (often advanced) cancer [3–7]. IWL can be an isolated sign of cancer, or can be associated with other cardinal signs (namely asthenia and anorexia) and/or with organ-specific signs and symptoms [2, 4]. Other known etiologies of IWL include many non-malignant organic diseases (accounting for 35–50% of cases, and mainly digestive system disorders) and psychiatric diseases (accounting for 15-30% of cases) [2–5].

Abnormal laboratory results are often described in patients with IWL and organic diseases [3–5, 7]. In this context, an elevated C-reactive protein (CRP) level and a high erythrocyte sedimentation rate (ESR) appears to be associated with a final diagnosis of cancer [3, 5, 7]. Inflammation is acknowledged to be a hallmark of cancer that substantially contributes to the development and progression of malignant disease [8]. Furthermore, systemic inflammation is part of a common definition of cancer cachexia [9]. In a large study of a cohort of patients with data on inflammatory biomarkers (including CRP, ESR, and plasma viscosity), the one-year cancer incidence [95% confidence interval (CI)] was 3.53% [3.37–3.70] in patients with at least one elevated marker (*n* = 116,708) and 1.50% [1.43–1.58] in those with normal inflammatory markers (*n* = 38,868) (*p* < 0.001) [10]. However, inflammatory markers had a low sensitivity (Se) for the diagnosis of cancer, and normal results did not rule out the presence of cancer [10].

Several groups of researchers have investigated the value of blood testing for a panel of tumor (bio)markers (TMs) in patients with suspect symptoms, including IWL [11–13]. A TM has notably been defined as “any biological molecule produced either by a tumor cell itself or by a tissue of the body in response to tumor invasion that can be objectively measured in body fluids and tissues and used as an indicator of the tumor process” [14, 15]. TM levels are low under normal conditions but rise in the presence of underlying cancer and are typically considered to reflect the tumor mass [14, 15]. Some TMs are associated with a specific histological type of cancer in a given organ, while others are associated with several histological types in different organs [14, 15]. Blood TM assays provide several advantages; they are low-cost, minimally invasive, automated tests that allow the rapid analysis of large numbers of samples and produce quantitative results with standardized reference values [15]. However, the use of TM assays in clinical practice is restricted by several factors. TMs can lack both Se (particularly for early-stage tumors) and specificity (Sp). TM levels can be elevated in many physiological situations and with benign pathologies, which can prompt inappropriate additional investigations [16, 17]. Thus, the current guidelines recommend TM assays to assist with cancer staging, prognosis, monitoring of disease progression, and early detection of relapse after treatment [16, 18, 19]. Routine TM-based cancer screening of asymptomatic or low-risk individuals is not recommended [16, 18, 19]. Furthermore, the diagnostic relevance of TMs is increasingly subject to debate controversial and is limited to certain specific contexts– often in association with imaging (e.g. cancer antigen 15 − 3 (CA 15 − 3) for a suspected adnexal mass) [16, 18, 19].

Nevertheless, the extrinsic performance of a diagnostic test (i.e. positive and negative predictive values (PPV and NPV)) for a given disease varies with the latter’s prevalence in the target population [20]. Trapé et al. assessed serum levels of 8 TMs (i.e. carcinoembryonic antigen (CEA), carbohydrate antigen (CA) 19 − 9, soluble fragments of cytokeratin 19 (CYFRA 21 − 1), CA 15 − 3, CA 125, neuron-specific enolase (NSE), alpha-fetoprotein (AFP), and prostate-specific antigen (PSA)) in 606 patients with significant IWL but no other suspicious signs [13]. For the diagnosis of cancer, a positive test (above the upper boundary provided by the laboratory) of at least one TM had a Se of 91.6%, a Sp of 55.9%, a PPV of 39.1% and a NPV of 95.7% (19). In Molina et al.’s study, a large panel of TMs (including those mentioned above, plus tumor-associated glycoprotein 72 (TAG-72), and squamous cell carcinoma (SCC)) was measured in the serum of 2,711 patients admitted to an internal medicine department with suspected cancer [11]. For diagnosis of a malignant disease in the cohort, the set of TMs gave a Se of 67.4% (75.4% in the 1280 patients with epithelial tumors), a Sp of 97.6%, a PPV of 97% and an NPV of 71.6% [11]. The measurement of circulating TM levels might help to evaluate the risk of underlying cancer in symptomatic patients, orientate further investigations, and reduce the diagnostic delay [11–13]. In previous published studies, data on inflammatory biomarkers were limited [11–13].

The objectives of the present study were to assess the diagnostic performance of (i) a panel of standard TMs in patients hospitalized with significant IWL and elevated levels of inflammation biomarkers, and (ii) a combination of the TM panel and the finding of the computed tomography (CT) scan often performed in the initial workup.

## Methods

### Cohort study

We performed a retrospective single-center study in the internal medicine department of Amiens-Picardie University Medical Center (Amiens, France) between January 1st, 2015, and November 1st, 2021.

After reviewing the inpatients’ medical records, we included individuals with (i) significant IWL, (ii) elevated levels of inflammation biomarkers, and (iii) data for at least two of the following TMs: CEA, total PSA, AFP, CA 125, CA 15 − 3, CA 19 − 9, calcitonin, and NSE. Significant IWL was defined as a loss of 5% or more between the usual weight (as reported by the patient and/or notified in medical records at a previous consultation or hospital admission over the latest 6 months) and the weight measured on admission. Patients lacking these data in their medical records were not included.

Elevated levels of inflammation biomarkers included an elevated serum CRP level (> 5 mg/L) and at least one of the following: (i) a high ESR, (ii) abnormal plasma levels of other inflammation proteins (fibrinogen, haptoglobin, albumin, and ferritin), (iii) an inflammatory serum protein electrophoresis profile (SPE), and (iv) a WBC count ≥ 10,000/mm^3^.

We excluded patients with (i) WL that could be explained by a low-calorie diet, other intentional measures, or the side effects of treatment, (ii) a history of progressing cancer or a cancer in remission for less than 5 years, and (iii) less than one year of follow-up after discharge from hospital (i.e. loss to follow-up).

### Data collection and definitions

Demographic, clinical, laboratory, histological and radiological data were extracted from medical reports (DxCare ® software, Dedalus, France).

All laboratory analyzes were performed in the central laboratory at Amiens-Picardie University Medical Center (see Supplementary Table S1). Other laboratory parameters (including the WBC count, the CRP, ESR, ferritin, fibrinogen, albumin, haptoglobin, hemoglobin, lactate dehydrogenase levels, and SPE) were analyzed as part of the initial diagnostic work-up or on admission. The ESR data were interpreted according to Sox et al.’s criteria [21].

We considered the following to be signs suggestive of cancer: (i) clinical signs such as asthenia, anorexia, fever, excessive sweating, external blood loss, venous thrombosis, chronic pain, palpable lymph nodes, hepatosplenomegaly, the presence of a mass, and lung, digestive tract, ENT, skin, neurologic or rheumatologic symptoms; (ii) abnormal laboratory results, including abnormal blood counts, hypercalcemia, hyponatremia, abnormal liver enzyme levels, and monoclonal gammopathy; (iii) CT findings, including the presence of a mass, suspect secondary lesions, lymph nodes with a shortest dimension > 1 cm, effusion, and venous thrombosis (peripheral vein and/or pulmonary embolisms).

The final diagnosis was established by the physician in charge of the patient either during or after the hospital stay. The diagnosed condition was classified as either malignant cancer (i.e. a solid cancer or a hematologic cancer) or a non-malignant pathology (a bacterial, viral, parasitic or fungal infection, an autoimmune disease (AID), an endocrine disease, a digestive disease, a benign tumor, organ failure, a toxic or iatrogenic adverse event, or a psychiatric disease).

Solid tumors were staged according to the World Health Organization classification, and lymphomas were staged according to the Ann Arbor classification [22].

Computed tomography findings were considered to be either “relevant” if the etiological hypothesis formulated by the radiologist was consistent with the final diagnosis or “false positive” if at least one suspicious radiologic finding was noted for a patient ultimately not diagnosed with cancer.

Cachexia was defined according to Evans et al.’s criteria [23], namely IWL > 5% over the previous 12 months (or body mass index < 20 kg/m^2^), asthenia, anorexia (decreases in muscle and adipose tissue could not be reliably assessed retrospectively) and at least one of the following criteria: serum CRP level > 5 mg/L, serum albumin level < 32 g/L, and blood hemoglobin level < 12 g/dL.

### Endpoints

The primary endpoint was the diagnostic performance (cancer vs. non-malignant condition) of the TM panel. The secondary endpoint was the diagnostic performance of the TM panel combined with CT.

### Statistical analysis

The result of each TM assay was considered as a binary qualitative parameter (i.e. positive or negative, depending on our central laboratory’s usual threshold). Groups and subgroups of patients were established according to their positivity for TMs and the final diagnosis (i.e. cancer or not). Patients with 1 or more positive TM assays were included in the “TM-positive group”, while patients with no positive TM assays were included in the “TM-negative group”.

In a descriptive analysis, categorical variables were expressed as the frequency (percentage), and continuous variables were expressed as the mean ± standard deviation (SD) or the median [interquartile range (IQR)] (depending on the data distribution). The Shapiro-Wilk test was used to determine whether data were normally distributed. In bivariate analyses of groups and subgroups, continuous variables were compared using Student’s t-test or a Wilcoxon-Mann-Whitney test (depending on the data distribution). Categorical variables were compared using a chi-squared test or Fisher’s exact test, if required.

The diagnostic performance of the TM assays was quantified in terms of the Se, Sp, PPV, NPV, positive likelihood ratio (LR+), negative likelihood ratio (LR-), and odds ratio (OR). These metrics were determined for each TM individually and then for the TM panel as a whole. Each metric’s 95%CI was determined using the adjusted Wald method or Wilson method with a correction for continuity. The threshold for statistical significance was set to *p* < 0.05. All analyses were performed using XLSTAT software (version 2020.5, Addinsoft, France).

### Ethics

This study was conducted in compliance with French legislation and the Declaration of Helsinki regarding ethics principles for medical research involving human subjects. The approval of the study was not required by an institutional review board according to the current French legislation on non-interventional retrospective researches (Law n° 2012 − 300 of March 5, 2012 on research involving the human person revised on April 12, 2018). Patients have been informed of their right to object to the use of their data for the present study, and have given their informed consent. The data processing was in compliance with the reference methodology MR-004 of the Commission Nationale de l’Informatique et des Libertés. The project was registered under the reference PI2022_843_0122.

## Results

### Characteristics of the study population

Between January 1st, 2015, and November 1th, 2021, 5338 patients were admitted to the internal medicine department at Amiens-Picardie University Medical Center, and 160 of these met the inclusion criteria. Twenty-five of the 160 patients met at least one exclusion criteria, and so 135 were included in the final analysis.

#### Demographic and clinical data (Table 1)

**Table 1:** Characteristics of the study participants

		TM-positive group (*N* = 76)			TM-negative group (*N* = 59)			
	Total (*N* = 135)	Cancer (*N* = 41)	No cancer (*N* = 35)	p*	Cancer (*N* = 9)	No cancer (*N* = 50)	p**	p***
**Age** (years), med [IQR]	74 [62.1–80.8]	75.9 [69.4–81.6]	70.6 [54.1–78.7]	0.20	73 [62.5–76.2]	73 [57.7–79.6]	0.92	0.39
**Female**, n (%)	57 (42.5)	21 (51.2)	13 (37.1)	0.43	2 (22.2)	21 (42.0)	0.40	0.62
**BMI** (kg/m2), med [IQR]	23.3 [20.1–27.0]	23.3 [20.3–26.8]	23.3 [20.1–27.0]	1	23.9 [21.0-26.5]	23.2 [20.1–27.0]	1	1
**Weight loss** (%), med [IQR]	8.9 [6.8–15.0]	9.4 [6.9–15.1]	9.5 [6.5–17.8]	0.86	9.3 [5.6–14.6]	8.6 [6.9–14.7]	0.43	0.75
**Time interval** (*weeks*), med [IQR]	8 [4–19]	8 [4–12]	11 [4–14]	NA	4 [4–16]	8 [–24]	NA	0.64
**History of cancer**, n/N (%)								
Personal	19/135 (14.1)	9/41 (21.9)	4/35 (11.4)	0.35	2/9 (22.2)	4/50 (8.0)	NA	0.35
Family	72/118 (61.0)	19/35 (46.3)	26/32 (45.7)	0.63	4/8 (44.4)	23/43 (46.0)	1	1
**Exposure**, n/N (%)								
Tobacco	80/132 (60.6)	22/39 (53.7)	23/35 (65.7)	0.23	6/9 (66.7)	29/49 (58.0)	0.96	1
Alcohol	33/118 (28.1)	12/34 (29.3)	8/33 (22.9)	0.47	1/8 (11.1)	12/43 (24.0)	NA	0.94
**Suspicious clinical signs**, n/N (%)								
Asthenia	79/94 (84.0)	25/28 (61.0)	21/24 (60.0)	1	4/8 (44.4)	29/34 (58.0)	0.14	0.32
Fever	32/129 (24.8)	8/40 (19.5)	9/34 (25.7)	0.7	2/9 (22.2)	13/46 (26.0)	NA	0.73
Anorexia	58/86 (67.4)	23/28 (56.1)	15/21 (42.9)	0.59	4/9 (44.4)	16/28 (32.0)	0.78	**0.041**
Chronic pain	38/132 (28.8)	6/40 (14.6)	10/34 (28.6)	0.23	1/9 (11.1)	21/49 (42.0)	NA	0.09
Sweat	22/60 (36.7)	4/18 (9.8)	7/15 (20.0)	0.26	3/5 (33.3)	8/22 (16.0)	0.64	0.75
Lymph node	25/132 (18.9)	8/40 (19.5)	5/34 (14.3)	0.77	3/9 (33.3)	9/49 (18.0)	0.62	0.82
Mass	6/133 (4.5)	5/41 (12.2)	0/33	NA	1/9 (11.1)	0/50	NA	NA
Hepatomegaly	22/128 (17.2)	12/39 (29.3)	5/34 (14.3)	0.16	2/9 (22.2)	3/46 (6.0)	NA	**0.045**
Bleeding	7/129 (5.4)	3/40 (7.3)	1/33 (2.9)	NA	0/9	3/47 (6.0)	NA	NA
Thrombosis	7/135 (5.2)	2/41 (4.9)	1/35 (2.9)	NA	0/9	4/50 (8.0)	NA	NA
Specific organ signs								
ENT	8/135(6.0)	2/41 (4.9)	1/35 (2.9)	NA	0/9	5/50 (10.0)	NA	NA
Digestive	61/135 (53.0)	25/41 (61.0)	11/35 (31.4)	**0.01**	5/9 (55.6)	20/50 (40.0)	0.84	0.69
Pulmonary	39/135 (28.9)	11/41 (26.8)	13/35 (37.1)	0.47	5/9 (55.6)	10/50 (20.0)	0.42	0.55
Cutaneous	14/135 (10.4)	4/41 (9.8)	6/35 (17.1)	0.55	2/9 (22.2)	2/50 (4.0)	NA	NA
Neurologic	22/135 (16.3)	7/41 (17.1)	8/35 (22.9)	0.73	0/9	7/50 (14.0)	NA	0.31
Rheumatologic	39/135 (28.9)	4/41 (9.8)	16/35 (45.7)	**0.001**	0/9	19/50 (38.0)	NA	0.58
**Laboratory test results**, n/N (%)								
Hypercalcemia	10/135 (7.4)	5/41 (12.2)	0/35	NA	1/9 (11.1)	3/49 (6.1)	NA	0.89
Hyponatremia	27/135 (20.0)	10/41 (24.4)	4/35 (11.4)	0.23	2/9 (22.2)	11/48 (22.4)	NA	0.69
Renal insufficiency	30/135 (22.2)	12/41 (29.3)	10/35 (28.6)	1	2/9 (22.2)	6/48 (12.3)	NA	0.052
Liver function abnormalities	49/135 (36.3)	18/40 (43.9)	14/35 (40.0)	0.84	4/9 (44.4)	13/49 (26.5)	0.52	0.15

Legends BMI: body mass index

ENT: ear, nose and throat

Med: median, NA: not applicable

IQR: interquartile range

SPE: serum protein electrophoresis

*TM: tumor marker*

* TM-positive patients with cancer vs. TM-positive patients without cancer

** TM-negative patients with cancer vs. TM-negative patients without cancer

*** TM-positive patients vs. TM-negative patients

The median age was 75.3 [63.9–84.8] for the women (*n* = 57) and 72 [58.1–78.5] for the men (*n* = 68) (*p* = 0.028). Nineteen of the 135 patients (14.1%) had a history of cancer in remission (> 5 years). Twenty-seven patients (20%) had a body mass index < 20 kg/m^2^. The prevalence of other general signs and organ-related functional signs reported by the patients are summarized in table 1.

#### Laboratory test results (Table 2)

**Table 2:** Laboratory test results

		TM-positive group (*N* = 76)			TM-negative group (*N* = 59)			
	Total (*N* = 135)	Cancer (*N* = 41)	No cancer (*N* = 35)	p*	Cancer (*N* = 9)	No cancer (*N* = 50)	p**	p***
**Inflammatory markers**, n^#^/N (%) med [IQR]								
CRP, mg/L *(N<5.0)*	135/135 (100) 78.8 [35.3-141.4]	78.6 [44.7–124.0]	52.7 [25.7-149.5]	0.87	134.0 [67.9–172.0]	81.4 [35.1-139.1]	0.17	0.67
ESR^§^, mm	40/135 (29.6) 61 [34.8–85.5]	11/41 (26.8) 58.0 [30.5–79.5]	8/35 (22.9) 70.0 [37.5–84.0]	0.89 0.39	3/9 (33.3) 79.5 [24 0.5-129.3]	18/50 (36.0) 63.0 [49.5–81.8]	1 0.11	0.25 0.39
Fibrinogen, g/L *(N:1.8–3.5)*	77/135 (57) 5.4 [4.2–6.8]	24/41 (58.5) 5.3 [4.6–6.7]	16/35 (45.7) 4.9 [3.6–6.2]	0.37 **0.04**	5/9 (55.3) 6.9 [3.9–8.2]	32/50 (64.0) 5.7 [4.2–2.1]	0.92 0.15	0.31 0.17
Haptoglobin, g/L *(N:0.4–2.8)*	25/41 (61.0) 2.6 [1.4–4.1]	8/13 (61.5) 3.2 [1.7–3.6]	2/8 (25.0) 1.4 [0.2–1.9]	NA **< 1.10** ^**− 4**^	2/2 (100) 5.4 [5.1–5.6]	13/18 (72.2) 2.6 [2.1–4.2]	NA 0.39	0.12 0.15
Ferritin, µg/l *(N:10–241)*	73/115 (63.4) 363.5 [157.5-706.5]	18/33 (54.6) 379 [137–558]	22/32 (68.8) 383 [240–692]	0.35 0.54	6/8 (75.0) 736.5 [289.5-953.5]	27/42 (64.3) 331.0 [153.7-672.3]	0.85 **0.03**	0.77 0.32
Albumin, g/L *(N:32–46)*	89/134 (66.4) 28.1 [25-34.1]	30/40 (75.0) 27.9 [24.9–31.9]	22/35 (62.9) 28.2 [25.4–34.6]	0.38 0.39	7/9 (77.8) 25.6 [23.5–30.2]	30/50 (60.0) 29. [24.0-35.4]	0.47 0.13	0.54 0.73
Inflammatory profile on SPE	69/117 (59.0)	15/27 (38.5)	17/33 (48.6)	0.96	6/9 (66 0.7)	31/48 (62.0)	**1**	0.27
**Other laboratory variables**, n/N (%) med [IQR]								
WBCs, 10^3^/mm^3^*(N:4.0–10.0)*	47/135 (34.8) 8.1 [6.2–11.3]	16/41 (39.0) 8.5 [6.8–12.0]	13/35 (37.1) 7.9 [5.7–13.8]	1 0.59	4/9 (44.4) 10.3 [7.8–15.7]	14/50 (28.0) 7.9 [6.2–10.3]	0.58 **0.01**	0.45 0.26
Hemoglobin, g/dL *(N:11.5–16)*	69/135 (51.1) 11.4 [9.9–12.4]	22/41 (53.7) 11 [9.4–12.1]	21/35 (60.0) 11.4 [9.9–12.4]	0.75 1	4/9 (44.4) 11.5 [10.0-12.6]	22/50 (44.0) 11.5 [10.4–12.3]	1 0.89	0.27 0.43
Platelets, 10^3^/mm3 *(N:150–400)*	40/135 (29.6) 290 [226.5–431)	10/41 (24.4) 278.5 [230.8-396.5]	10/35 (28.6) 520 [469.3-615.5]	0.88 0.83	1/9 (11.1) 254.0 [241.3-294.5]	19/50 (38.0) 304.5 [237.0-463.5]	NA 0.22	0.45 0.46
LDH, U/L *(N:120–246)*	102/107 (95.3) 660 [354–602]	31/32 (96.9) 520.5 [382.7-746.5]	23/25 (92.00) 435.0 [354–582]	0.83 NA	7/7 (100) 341 [308–593]	41/43 (95.4) 414 [351–560]	1 NA	1 1
**Blood TMs**, n^#^ /N (%) med [IQR]								
CEA, µg/L *(N < 2.5-5)*	28/128 (21.9) 1.5 [0.9-3.0]	17/40 (42.5) 2.7 [1.0–6.0]	11/35 (31.4) 1.7 [1.2–3.9]	0.06 NA	0/8 1.5 [1.0-2.2]	0/45 1.1 [0.8–1.9]	NA NA	NA NA
Total PSA, ng/mL *(N < 4)*	10/66 (13.3) 1.4 [0.6–2.8]	4/17(23.5 )1.4 [0.7–3.6]	6/19 (31.6) 2.4 [1.2–4.6]	0.60 NA	0/5 0.7 [0.4–1.6]	0/25 1.2 [0.5–1.9]	NA NA	NA NA
AFP, ng/mL *(N < 8)*	7/118 (5.9) 3.0 [1.9–4.5]	5/35 (14.3) 3.5 [2.4–4.6]	2/31 (6.5) 3.3 [2.1–4.2]	NA NA	0/7 3.3 [1.7–4.4]	0/45 2.2 [1.7–3.5]	NA NA	NA NA
CA 125, U/mL *(N < 35)*	38/96 (39.6) 18.0 [9.6–61.9]	22/31 (71.0) 124.5 [28.6-210.9]	16/27 (59.3) 38.4 [11.9–57.3]	0.51 NA	0/6 15.0 [9.2–17.8]	0/32 9.5 [6.7–13.1]	NA NA	NA NA
CA 15 − 3, U/mL *(N < 32.4)*	17/91 (18.7) 17.4 [11-26.4]	11/30 (36.7) 25.7 (17.3–65.9]	6/25 (24.0) 17.7 [11.2–31.5]	0.46 NA	0/5 11.2 [10.9–15.2]	0/31 12.60 [7.5–17.8]	NA NA	NA NA
CA 19 − 9, U/mL *(N < 37)*	19/121(15.7) 11.6 [7.8–21.7]	15/34 (44.1) 19.5 [8.9-218.9]	4/34 (11.8) 13.9 [8.4–28.4]	**0.004** NA	0/9 9.7 [5.3–10.1]	0/44 10.1 [6.4–14.1]	NA NA	NA NA
Calcitonin, pg/mL *(N < 10)*	6/55 (10.9) 4.4 [2.8–10.8]	1/17 (5.9) 4.3 [3.2–5.9]	5/17 (29.4) 15.0 [9.0-19.5]	NA NA	0/3 3.8 [3.5–4.2]	0/18 1.6 [0.9–3.6]	NA NA	NA NA
NSE, µg/L *(N < 18.3)*	19/63 (30.2) 12 [9.8–22]	12/22 (54.6) 22.4 [10.3–45.7]	7/20 (35.0) 12.2 [9.7–20.4]	0.33 NA	0/4 10.3 [9.8–12.1]	0/17 10.8 [8.5–13.8]	NA NA	NA NA

Legends CRP: C-reactive protein

ESR: erythrocyte sedimentation rate

LDH: lactate dehydrogenase, NA: not applicable

IQR: interquartile range

SPE: serum protein electrophoresis

TM: tumor marker

WBC: white blood cell

^#^ number of patients with a value above the upper normal boundary set by the central laboratory (except for albumin and hemoglobin, where the value was below the lower normal boundary)

^§^ according to *Sox et al.* (Sox and Liang 1986)

* TM-positive patients with cancer vs. TM-positive patients without cancer

** TM-negative patients with cancer vs. TM-negative patients without cancer

*** TM-positive patients vs. TM-negative patients

The median CRP value was 78.8 [35.3-141.1] mg/L. An abnormally high lactate dehydrogenase level (> 246 U/L) was observed in 102 (95%) of the 107 patients with data. Thirty-four patients (25.2%) met the criteria for cachexia.

Seventy-six patients (56.3%) had at least one positive TM assay. Of the 76 patients in the TM-positive group, 33 had a single positive marker, 25 had two positive markers, and 18 patients had three or more positive markers.

#### CT findings (Table 3)

**Table 3:** Computed tomography findings

		TM-positive group (*N* = 76)			TM-negative group (*N* = 59)			
	Total (*N* = 135)	Cancer (*N* = 41)	No cancer (*N* = 35)	p*	Cancer (*N* = 9)	No cancer (*N* = 50)	p**	p***
**CT**, n/N(%)	131/135 (97.0)	41/41 (100)	35/35 (100)	1	9/9 (100)	46/50 (92.0)	0.71	0.11
**Region**, n/N(%)								
Cerebral	22/131 (16.8)	10/41 (24.4)	7/35 (20.0)	0.86	2/9 (22.2)	3/46 (6.5)	NA	0.06
Cervical	17/131 (13.0)	5/41 (12.2)	3/35 (8.6)	0.89	1/9 (11.1)	8/46 (17.4)	NA	0.48
Thoracic	114/131 (87.0)	35/41 (95.1)	30/35 (85.7)	1	7/9 (77.8)	42/46 (91.3)	0.63	0.73
Abdomen and pelvis	113/131 (86.3)	39/41 (95.1)	26/35 (74.3)	**0.03**	9/9 (100.0)	40/46 (87.0)	0.20	0.73
**Suspicious findings**, n/N(%)	52/131 (39.7)	36/41 (87.8)	3/35 (8.57)	**< 1.10** ^**− 4**^	7/9 (77.8)	6/46 (13.0)	1	**0.008**
Mass, n/N(%)	40/131 (30.5)	26/41 (63.4)	3/35 (8.6)	**< 1.10** ^**− 4**^	7/9 (77.8)	4/46 (8.7)	**< 1.10** ^**− 4**^	**0.032**
Suspicious secondary lesion(s), n/N(%)	27/131 (20.6)	21/41 (51.2)	0	NA	4/9 (44.4)	2/46 (4.3)	NA	**0.021**
Enlarged lymph node(s), n/N(%)	56/131 (42.8)	26/41 (63.4)	11/35 (31.43)	**0.003**	6/9 (66.7)	13/46 (28.3)	0.06	0.11
Effusion, n/N(%)	40/131 (30.5)	19/41 (46.3)	9/35 (25.7)	0.06	3/9 (33.3)	9/46 (19.6)	0.69	0.08
Thrombosis, n/N(%)	15/131 (11.5)	8/41 (19.5)	2/35 (5.7)	NA	2/9 (22.2)	3/46 (6.5)	NA	0.63
**Relevant for final diagnosis**, n/N(%)	62/131 (47.3)	36/41 (87.8)	8/35 (22.9)	**< 10** ^**− 4**^	7/9 (77.8)	11/46 (23.9)	**< 1.10** ^**− 4**^	**< 1.10** ^**− 4**^
**False positive**, n/N(%)	9/131 (6.9)	0/41	3/35 (8.6)	NA	0/9	6/46 (13.0)	NA	0.26

*Legends: CT: computed tomography, NA: not applicable*

* TM-positive patients with cancer vs. TM-positive patients without cancer

** TM-negative patients with cancer vs. TM-negative patients without cancer

*** TM-positive patients vs. TM-negative patients

One hundred and thirty-one (97%) of the 135 patients had undergone a CT scan. Fifty-two patients (39.7%) had signs of possible cancer on the CT. The CT findings were considered to be “relevant” for the final diagnosis of 62 patients (47.3%) and “false positive” for 9 patients (6.9%).

#### Final diagnosis (Table 4)

**Table 4:** Final diagnosis

		TM-positive group (*N* = 76)		TM-negative group (*N* = 59)		
	Total (*N* = 135)	Cancer (*N* = 41)	No cancer (*N* = 35)	Cancer (*N* = 9)	No cancer (*N* = 50)	p*
**Cancer**, n/N (%)	50/135 (37.0)	41/76 (53.9)	0/76	9/59 (15.3)	0/59	**< 1.10** ^**-4**^
**Organ**, n/N (%)						
Hematologic cancer	12/50 (24.0)	9/41 (22.0)		3/9 (33.3)		0.79
Upper digestive tract^#^	3/50 (6.0)	2/41 (4.9)		1/9 (11.1)		NA
Lower digestive tract^§^	5/50 (10.0)	3/41 (7.3)		2/9 (22.2)		NA
Liver	2 /50 (4.0)	2/41 (5.0)		0/9		NA
Pancreas	3/50 (6.0)	3/41 (7.3)		0/9		NA
Biliary tract	3/50 (6.0)	3/41 (7.3)		0/9		NA
Lung	7/50 (14.0)	6/41 (14.6)		1/9 (11.1)		NA
Breast	3/50 (6.0)	3/41 (7.3)		0/9		NA
Ovary	3/50 (6.0)	3/41 (7.3)		1/9 (11.1)		NA
Prostate	2/50 (4.0)	1/41 (2.4)		1/9 (11.1)		NA
Urinary tract	2/50 (4.0)	1/41 (2.4)		0/9		NA
Central nervous system	1/50 (2.0)	1/41 (2.4)		0/9		NA
Not known or uncertain	4 /50 (8.0)	4/41 (9.8)		0/9		NA
**Histology**, n/N(%)						
Epithelial tumor	32/47 (68.1)	26/38(68.4)		6/9 (66.67)		1
Nonepithelial tumor	15/47(31.9)	12/38 (31.6)		3/9 (33.33)		1
- *Neuroendocrine tumor*	2/15 (12.5)	2/12 (16.7)		0/9		NA
- *MPS*	9/15 (56.3)	6/12 (50.0)		3/3 (100)		**0.043**
- *LPS*	1/15 (6.3)	1/12 (8.3)		0/3		NA
- *MDS*	2/15 (12.5)	2/12 (16.7)		0/3		NA
- *GIST*	1/15 (6.3)	1/12 (8.3)		0/3		NA
**Stage**, n/N(%)						
I	5/42 (11.9)	3/33 (9.1)		2/9 (22.2)		NA
II	4/42 (9.5)	2/33 (6.1)		2/9 (22.2)		NA
III	2/42(4.8)	2/33 (6.1)		0/9		NA
IV	31/42 (73.8)	26/33 (78.8)		5/9 (55.6)		0.37
**Benign disease**, n/N(%)	81/135 (60.0)		35/76 (46.0)		46/59(78.0)	**< 0.001**
Infection	14/81 (17.3)		5/35 (14.3)		9/46 (19.6)	0.74
AID	45/81 (55.6)		22/35 (62.8)		23/46 (50)	0.35
Endocrinopathy	2/81(2.5)		1/35 (2.9)		1/46 (2.2)	NA
Digestive tract disorder	7/81 (8.6)		2/35 (5.7)		5/46 (10.9)	NA
Neurologic disorder	3/81 (3.7)		1/35 (2.9)		2/46 (4.3)	NA
Crystal arthropathy	2/81 (2.5)		2/35 (5.7)		0/46	NA
Adverse drug reactions	2/81(2.5)		0/35		2/46 (4.3)	NA
Other	6/81 (7.4)		2/35 (5.7)		4/46 (8.7)	NA
**No final diagnosis**, n/N(%)	4/135 (3.0)		0/35		4/50 (8.0)	NA

*Legends AID: autoimmune disease, GIST: gastrointestinal stromal tumor, LPS: lymphoproliferative syndrome, MDS: myelodysplasia syndrome, MPS: myeloproliferative syndrome, NA: not applicable*

* TM-positive patients vs. TM-negative patients

A final diagnosis was established during the hospital stay or within 12 months of discharge for 131 (97.0%) of the 135 patients. The four patients (3.0%) without a final diagnosis had received a comprehensive check-up and were followed up for between 18 and 36 months.

Cancer was found in 50 patients (37.0%), with 34 cases of solid cancer and 12 cases of hematologic cancer. Most of the patients with staging data (31 out of 42) were diagnosed with late-stage cancer (stage IV). The diagnosis was confirmed histologically in 47 of the 50 patients (94%). For the three other patients, the diagnosis of cancer was supported by imaging findings: peritoneal carcinosis (*n* = 1), a brain tumor meeting the radiologic criteria for diffuse glioma (*n* = 1), and liver metastases (*n* = 1).

Among the 81 patients without cancer, 45 had an AID (55.6%; mostly vasculitis (*n* = 16) or polymyalgia rheumatica (*n* = 12)).

Final diagnoses based on each positive tumor marker are detailed in Supplementary Table S2.

#### Comparisons of patients with cancer and those without

The patients with cancer and those without did not differ significantly with regard to the demographic data. Patients with cancer were significantly more likely to have digestive signs (30 out of 50, vs. 31 of the 85 patients without cancer; *p* = 0.011), including hepatomegaly (14 out of 48 vs. 8 out of 80, respectively; *p* = 0.018). In contrast, patients without cancer were significantly more likely to have chronic pain (31 out of 85, vs. 6 of the 48 patients with cancer, respectively; *p* < 0.002) and rheumatologic signs (35 out of 85 vs. 4 out of 50, respectively; *p* < 10^− 4^). There were no significant intergroup differences in the other clinical and laboratory parameters.

### Diagnostic performances of the TM panel for cancer

A cancer was diagnosed in 41 (53.9%) of the 76 patients in the TM-positive group and in 9 (15.2%) of the 59 patients in the TM-negative group (*p* < 10^− 4^) (Table 4). The performances of each TM for the diagnosis of cancer are detailed in Supplementary Table S3. The diagnostic performance was not altered in patients with liver and kidney function disorders (results not shown) but CA 125 was less specific in patients with effusions (Sp: 0.36 [0.15–0.65]).

One or more positive TM assays had a Se of 0.80 [0.66–0.90], a Sp of 0.61 [0.50–0.71], a PPV of 0.55 [0.43–0.66], an NPV of 0.84 [0.75–0.93], a LR + of 2.06 [1.53–2.78] and a LR- of 0.33 [0.18–0.58] for the diagnosis of cancer.

The exclusion of NSE and calcitonin from the TM panel did not markedly modify the value of one or more positive TM assays for the diagnosis of epithelial tumors (Se: 0.84 [0.67–0.93], Sp: 0.59 [0.49–0.68], PPV: 0.38 [0.26–0.49], NPV: 0.92 [0.86–0.99], LR+: 2.03 [1.54–2.67], LR-: 0.28 [0.12–0.62], OR: 7.38 [2.72–19.99]).

Twenty (35.3%) of the 34 patients with cachexia had cancer. In this subgroup, the full TM panel had a Se of 0.86 [0.59–0.97], a Sp of 0.50 [0.30–0.70], a PPV of 0.56 [0.34–0.75], an NPV of 0.83 [0.62-1.0], a LR + of 1.71 [1.05–2.8], and a LR- of 0.29 [0.07–1.11] (OR of 6.0 [1.2–29.9]) for the diagnosis of cancer.

### Diagnostic performance of the TM panel when combined with CT

#### Diagnostic relevance of CT

CT was considered to be more relevant for the final diagnosis in patients with cancer than in those without (*p* < 10^− 4^) (Table 3). Patients with cancer were significantly more likely to have a mass (33 out of 50, vs. 7 of the 81 patients without cancer, respectively; *p* < 10^− 4^), enlarged lymph nodes (32 out of 50, vs. 24 out of 81, respectively; *p* < 10^− 4^), and effusion (22 out of 50 vs. 18 out of 81, respectively; *p* = 0.012).

For five of the TM-positive patients with cancer (including two cases of myelodysplastic syndrome, one of myeloproliferative syndrome and one of intravascular lymphoma), a CT scan of the thorax, abdomen and pelvis did not identify any signs of malignancy.

#### Diagnostic performance of the TM panel when combined with CT

When combined with the CT findings, one or more positive TM assays had a Se of 0.81 [0.66–0.92], a Sp of 0.67 [0.35–0.88], a PPV of 0.92 [0.08–0.30], an NPV of 0.43 [0.17–0.69], a LR + of 2.43 [0.95–6.19], a LR- of 0.29 [0.13–0.62], and an OR of 8.5 [1.90–38.1] for the diagnosis of cancer. For patients with no suspect signs on CT, the TM panel’s NPV was 0.96 [0.89-1.00]. CT alone (i.e. regardless of the TM results) had a Se of 0.84 [0.71–0.92], a Sp of 0.89 [0.80–0.94], a PPV of 0.82 [0.72–0.93], an NPV of 0.90 [0.83–0.97], a LR + of 7.56 [4.04–14.16], an LR- of 0.18 [0.10–0.34], and an OR of 42.00 [15.45-114.15] for the diagnosis of cancer.

## Discussion

Involuntary weight loss is a frequent indication for admission to an internal medicine department [5–7]. A diagnosis of cancer can then be considered– especially when IWL is associated with elevated levels of blood markers of systemic inflammation. The patient’s medical history, clinical examination, routine laboratory results and even first-line medical imaging findings might not be able to discriminate reliably between a malignant or benign pathology. Blood tests of conventional TMs are still performed widely for diagnostic purposes in patients with alarming and possible cancer-related clinical symptoms and/or radiological findings, and a positive TM assay may prompt the physicians to prescribe additional examinations [16, 24]. However, published data on the relevance of TM testing in this context are scarce and subject to debate [11, 12].

Cancer was diagnosed in 50 (27%) of our 135 patients. Most of these cancers were late-stage. The patients with cancer and those without did not differ significantly in terms of demographics, clinical characteristics, or inflammatory biomarker levels. These data indicate that further investigations are required for a prompt, robust, final diagnosis. With regard to the TM panel in our study, each individual TM’s Se for cancer diagnosis was low. This poor performance was doubtless related to the small number of patients in some subgroups. For example, calcitonin is a specific marker of medullary thyroid cancer, and no such cases were diagnosed in our cohort [18]. Although prostate cancer is common in the general population in France (due to a national screening program and the long course of the disease), it was found in only two of our patients [25]. In our study, we excluded patients with a history of progressing cancer. Furthermore, certain cancers (including prostate cancer and breast cancer) appear to be less frequently associated with a systemic inflammatory response [10]. Nevertheless, ≥ 1 positive TMs gave a Se of 80% for the diagnosis of cancer, and the NPV was above 80% when all the TM assays were negative. Thus, a negative result for our TM panel would be reassuring– even in patients with cachexia.

Our present results also highlighted the TMs’ lack of Sp– even in a selected population– because ≥ 1 positive TM had a PPV for cancer diagnosis of only 55%. In a cohort of 606 patients with IWL but no data on inflammation markers, Trapé et al. reported an NPV of 96% but a PPV of only 39.1% for ≥ 1 out of 8 positive TMs (according to the threshold set by the laboratory) [13]. In routine clinical practice, cut-off values are determined with an emphasis on Se, so as not to miss a case of cancer [20]. Thus, in Molina et al.’s study of 2711 patients, the marker’s positivity thresholds were chosen to obtain greater Sp and were higher than those used in the present study. Although the PPV of Molina et al.’s panel was 97%, the NPV only 72% (due to a lower Se) [11]. Bosch et al. recently published the results of a study of 11 TMs (CEA, PSA, AFP, CA 125, CA 15 − 3, CA 19 − 9, NSE, TAG-72, CYFRA 21 − 1, SCC, and gastrin-releasing-peptide precursor) in 4776 patients with suspicious clinical signs (fever, IWL, pain, mass, externalized bleeding, thrombosis, skin lesions, and pulmonary, digestive or neurologic symptoms), abnormal laboratory test results (anemia and other unspecified abnormalities) or radiological findings (fractures and bone lesions) [12]. In order to achieve a Sp ≥ 95% for each TM, the upper boundaries were adjusted by taking account of the presence of renal failure, liver disease, effusions, and skin lesions. Thus, for the diagnosis of a malignant epithelial tumor in this large cohort (*n* = 1,214), the overall Se (≥ 1 positive TMs) was 72.2%, with a Sp of 98%, a PPV of 93%, and an NPV of 90.5% [12]. Given the small size of our study population, we were unable to estimate an alternative cut-off value for each TM and that might have been more relevant in clinical practice.

A high proportion (55.6%) of the patients without cancer had an AID. There are several rationales for TM assays in these patients: (i) the clinical presentation of AID is quite variable, and weight loss can be frequent and significant at the time of diagnosis (i.e. when the disease is active and untreated) [26, 27], (ii) some AIDs (e.g. sarcoidosis, or granulomatosis with polyangiitis) can mimic tumors [27, 28], (iii) some AIDs (e.g. myositis) are associated with an elevated risk of cancer, and (iv) some cancers have autoimmune manifestations [29].

Our analysis of the diagnosis value of CT in cancer indicates that this examination is relevant for identifying malignant disease in patients with significant IWL and systemic inflammation. Most cases of cancer were diagnosed at an advanced stage, and CT frequently found a mass, enlarged lymph nodes or possible metastases. Goh et al. studied the relevance of CT for the investigation of significant IWL (*n* = 200 patients); 56 patients (including 42 ultimately diagnosed with cancer) had highly suspicious signs of malignancy on the CT scan and 123 (including only two ultimately diagnosed with cancer) had a negative CT scan [30]. In Goh et al.’s study, CT had a Se of 95%, a Sp of 77%, a PPV of 55%, and an NPV of 98.4% for the diagnosis of cancer [30]. In our study, the PPV of CT alone (i.e. independently of the TM results) was 82%, and the NPV was 90%. The combination of suspicious CT findings with one or more positive TM assays gave a PPV of 92%, whereas the combination of a negative CT scan with a fully negative TM panel had an NPV of 96%. However, the CT scan did not find suspicious signs in several patients ultimately diagnosed with hematologic cancer. Like the AIDs, some hematologic cancers have a broad range of presentations and can be associated with systemic inflammation [31, 32]. A bone marrow biopsy should therefore be considered in patients with IWL and elevated blood levels of inflammation biomarkers but no suspicious signs on CT.

Lastly, our results highlighted the limitations of a standard TM panel and CT for the detection of certain malignant cancers. Researchers are now turning to non-protein TMs (e.g. tumor DNA or mRNA) that might be detectable in biological fluids and tissue biopsies and might enable cancer to be diagnosed even earlier [33]. In addition, fluorodeoxyglucose positron emission tomography (FDG-PET)/CT is essential for the management of most types of cancer according to clinical guidelines, although it is not recommended for the suspicion of occult neoplastic disease. However, FDG PET/CT is approved for patients whose primary tumor is unknown [34]. Similarly, García Vicente et al. indicated its potential benefit for paraneoplastic neurological syndrome [35]. Finally, in patients with significant IWL and high levels of inflammation biomarkers, AID such as vasculitis and polymyalgia rheumatica are common differential diagnoses. FDG PET/CT can also aid in diagnosing these conditions [36].

Our study had several limitations. Firstly, our strict inclusion criteria and missing data for some variables meant that the sample size was small. However, we only included patients with well documented WL in medical records; in larger cohorts, the inclusion criteria for WL tend to be less strict (e.g. WL over a longer (12-month) period, WL reported but not quantified, a change in clothing size, and interviews with family members). Secondly, our TM panel was restricted to conventional TMs assayed routinely in our central laboratory; some earlier studies assessed more TMs than we did. Thirdly, not all of our patients had undergone a complete set of TM assays and/or a CT scan of the thorax, abdomen and pelvis or the whole body. Fourthly, in some cases, the final diagnosis noted in the medical records did not account for the high observed levels of inflammation biomarkers, such as drug adverse reactions.

## Conclusion

In patients with IWL and high levels of inflammation biomarkers observed, a blood TM panel (including CEA, total PSA, AFP, CA 125, CA 15 − 3, CA 19 − 9, calcitonin and NSE) appears to be of moderate diagnostic value for discriminating between benign and malignant diseases. However, the absence of a positive TM test suggested that cancer was unlikely– especially when no suspicious features had been observed on a CT scan.

### Electronic supplementary material

Below is the link to the electronic supplementary material.

Supplementary Material 1

## Data Availability

The data that support the findings of this study are available on request from the corresponding author. The data are not publicly available, due to privacy or ethical restrictions.

## Abbreviations

AFP: Alpha-Fetoprotein
AID: Autoimmune Disease
CA: Carbohydrate Antigen
CEA: Carcinoembryonic Antigen
CT: Computed Tomography
CRP: C-Reactive Protein
ESR: Erythrocyte Sedimentation Rate
FDG PET: Fluorodeoxyglucose Positron Emission Tomography
IWL: Involuntary Weight Loss
NPV: Negative Predictive Value
NSE: Neuron-Specific Enolase
PPV: Positive Predictive Value
PSA: Prostate-Specific Antigen
Se: Sensitivity
SPE: Serum Protein Electrophoresis
CYFRA 21-1: Soluble Fragments of Cytokeratin 19
Sp: Specificity
SCC: Squamous Cell Carcinoma
TM: Tumor Marker
TAG-72: Tumor-Associated Glycoprotein 72
WBC: White Blood Cell
