# Supplementary material for: Relevance of blood tumor markers in inpatients with significant involuntary weight loss and elevated levels of inflammation biomarkers
Source: BMC Cancer. 2024 Apr 15;24:468. doi: 10.1186/s12885-024-12201-0 (PMC11017702; doi:10.1186/s12885-024-12201-0)
Supplement: Supplementary file 1 — Supplementary Material 1 [file 12885_2024_12201_MOESM1_ESM.docx]

| **Tumor marker** | **Laboratory standards**  *before 2019*  from 2019 | **Sample** | **Analysis method** | **Analyzer and** **manufacturer**  *before 2019*  from 2019 |
| --- | --- | --- | --- | --- |
| **CEA** | *<3 ng/mL*  <2.5 µg/L | Serum | Sandwich chemiluminescent immunoassay | *LOCI Vista® 500 Siemens*  Siemens Atellica |
| **Total PSA** | *<4 ng/mL*  <4 ng/mL |  |  | *LOCI Vista® 500 Siemens*  Siemens Atellica® IM1600 |
| **AFP** | *<8 ng/mL*  <8 ng/mL |  |  | *LOCI Vista® 500 Siemens*  Test Atellica^TM^ IM |
| **CA 125** | *<35 U/mL*  <35U/mL |  |  | *Centaur® XP Siemens*  Test Atellica^TM^ IM |
| **CA 15-3** | *<23 U/mL*  <32.4 U/mL |  |  | *Centaur® XP Siemens*  Test Atellica^TM^ IM |
| **CA 19-9** | *<35 U/mL*  <37 U/mL |  |  | *Centaur® XP Siemens*  Test Atellica^TM^ IM |
| **Calcitonin** | *<10 pg/mL*  <10 pg/mL |  |  | *Liaison XL Diasorin*  Liaison XL Diasorin |
| **NSE** | *<18.3 µg/L*  <18.3 µg/L |  |  | *Liaison XL Diasorin*  Liaison XL Diasorin |

**Table S1: Blood tests for tumor markers conducted at the Human Biology Laboratory of Amiens-Picardie University Hospital**

**Table S2: Final diagnoses of patients in the tumor marker positive group according to each tumor marker**

|  | **CEA** | **Total PSA** | **AFP** | **CA 125** | **CA 15-3** | **CA 19-9** | **Calcitonin** | **NSE** |
| --- | --- | --- | --- | --- | --- | --- | --- | --- |
| **Positive TM**, n/N (%) | 28/128 (21.9) | 10/66 (15.2) | 7/118 (5.9) | 38/96 (39.6) | 17/91 (18.7) | 19/121 (15.7) | 6/55 (10.9) | 19/63 (30.2) |
| **Number of associated positive TMs**, n/N (%) |  |  |  |  |  |  |  |  |
| 1 | 9(32.1) | 4/10 (40.0) | 3/7 (42.9) | 7/38 (18.4) | 1/17 (5.9) | 1/19 (5.2) | 3/6 (50) | 5/19 (26.3) |
| 2 | 11(39.3) | 4/10 (40.0) | 1/7 (14.3) | 13/38 (34.2) | 4/17 (23.5) | 7/19 (36.8) | 1/6 (16.7) | 8/19 (42.1) |
| 3 | 3(10.7) | 1/10 (10.0) | 0 | 10/38 (26.3) | 6/17 (35.3) | 6/19 (31.6) | 0 | 4/19 (21.1) |
| 4 | 5 (17.8) | 1/10(10.0) | 2/7 (28.6) | 7/38 (18.4) | 5/17 (29.4) | 4/19 (21.1) | 2/6 (33.3) | 2/19 (10.5) |
| 5 | 0 | 0 | 1/7 (14.3) | 1/38 (2.6) | 1/17 (5.9) | 1/19 (5.2) | 0 | 0 |
| **Cancer**, n/N (%) | 17/28(60.7) | 4/10(40.0) | 5/7(71.4) | 22/38(57.9) | 11/17(64.7) | 15/19(78.9) | 1/6 (16.7) | 12/19 (63.2) |
| **Organ***,* n/N(%) |  |  |  |  |  |  |  |  |
| Hematologic cancer | 3/17 (17.6) | 0 | 1/5 (20) | 5/22 (22.7) | 0 | 1/15 (6.7) | NA | 5/12 (41.7) |
| Upper digestive tract^#^ | 0 | 1/3(33.3) | 1/5 (20) | 0 | 0 | 0 |  | 0 |
| Lower digestive tract^§^ | 3/17 (17.6) | 1/3 (33.3) | 0 | 0 | 0 | 2/15(13.3) |  | 0 |
| Liver | 1/17 (5.9) | 0 | 1(20) | 2/22 (9.1) | 1/11 (9.1) | 2/15 (13.3) |  | 0 |
| Pancreas | 1/17 (5.9) | 0 | 0 | 3/22 (13.6) | 1/11 (9.1) | 3/15 (20) |  | 1/12 (8.3) |
| Biliary tract | 0 | 0 | 1(20) | 3/22 (13.6) | 1/11 (9.1) | 1/15 (6.7) |  | 1/12 (8.3) |
| Lung | 2/17 (11.8) | 0 | 1(20) | 2/22 (9.1) | 3/11 (27.3) | 1/15 (6.7) |  | 2/12 (16.7) |
| Breast | 3/17 (17.6) | 0 | 0 | 1/22 (4.54) | 2/11 (18.2) | 0 |  | 1/12 (8.3) |
| Ovary | 1/17 (5.9) | 0 | 0 | 3/22 (13.6) | 1/11 (9.1) | 2/15 (13.3) |  | 0 |
| Prostate | 0 | 1/3 (33.3) | 0 | 0 | 0 | 0 |  | 0 |
| Urinary tract | 1/17(5.9) | 0 | 0 | 1/22 (4.54) | 1/11 (9.1) | 1/15 (6.7) |  | 0 |
| Central nervous system | 1/17 (5.9) | 0 | 0 | 1/22 (4.54) | 0 | 1/15 (6.7) |  | 0 |
| Not known or uncertain | 1/17 (5.9) | 0 | 0 | 1/22 (4.54) | 1/11 (9.1) | 1/15 (6.7) |  | 2/12 (16.7) |
| **Histology**, n/N(%) |  |  |  |  |  |  |  |  |
| Epithelial tumor | 13/16 (81.3) | 3/3 (100) | 3/5 (60.0) | 14/20 (70.0) | 9/10 (90.0) | 12/13 (92.3) | NA | 5/12 (41.7) |
| Nonepithelial tumor | 3/16 (18.8) | 0 | 2/5 (40.0) | 6/20 -30.0) | 1/10 (10.0) | 1/13 (7.7) |  | 7/12 (58.3) |
| - *Neuroendocrine tumor* | 0 |  | 0 | 1/20 (5.0) | 1/10 (10.0) | 0 |  | 2/12 (16.7) |
| - *MPS* | 1/16 (6.3) |  | 1/5 (20.0) | 5/20 (25.0) | 0 | 1/13 (7.7) |  | 4/12 (33.3) |
| - *LPS* | 0 |  | 0 | 0 | 0 | 0 |  | 1/12 (8.7) |
| - *MDS* | 2/16 (12.5) |  | 0 | 0 | 0 | 0 |  | 0 |
| - *GIST* | 0 |  | 1/5 (20.0) | 0 | 0 | 0 |  | 0 |
| **Stage**, n/N(%) |  |  |  |  |  |  |  |  |
| I | 2/14(14.3) | 1/3 (33.3) | 1/4 (25.0) | 1/20 (5.0) | 0 | 1/14 (7.1) | NA | 0 |
| II | 1/14(7.1) | 1/3(33.3) | 1/4 (25.0) | 1/20 (5.0) | 0 | 1/14 (7.1) | NA | 0 |
| III | 0 | 0 | 0 | 1/20 (5.0) | 1/11 (9.1) | 0 | NA | 0 |
| IV | 10/14 (71.4) | 1/3 (33.3) | 2/4 (50.0) | 17/20 (85.0) | 10/11 (90.9) | 12/14 (85.8) | 01-janv | 9/9 (100) |
| **Benign disease**, n/N(%) | 11/28 (39.3) | 6/10 (60.0) | 2/7 (28.6) | 16/38 (42.1) | 6/17 (35.3) | 2/19 (10.5) | 5/6 (83.3) | 7/19 (36.8 |
| Infection | 2/11 (18.2) | 1/6 (16.7) | 0 | 4/16 (25.0) | 1/6 (16.7) | 1/4 (25.0) | 0 | 1/7 (14.3) |
| AID | 7/11 (63.6) | 4/6 (66.7) | 1/2 (50.0) | 8/16 (50) | 5/6 (83.3) | 2/4 (50.0) | 5/5 (100) | 5/7 (71.4) |
| Endocrinopathy | 1/11 (9.1) | 0 | 0 | 0 | 0 | 0 | 0 | 0 |
| Digestive tract disorder | 1/11 (9.1) | 0 | 0 | 1/16 (6.3) | 0 | 0 | 0 | 0 |
| Neurologic disorder | 0 | 0 | 0 | 1/16 (6.3) | 0 | 0 | 0 | 0 |
| Crystal arthropathy | 0 | 0 | 0 | 1/16 (6.3) | 0 | 0 | 0 | 1/7 (14.3) |
| Other | 0 | 1/6 (16.7) | 1/2 (50.0) | 1/16 (6.3) | 0 | 1/4 (25.0) | 0 | 0 |

*Legends: AI: autoimmune disease, CNS: central nervous system, GIST: gastrointestinal stromal tumors, HIV: human immunodeficiency virus, NET: neuroendocrine tumor, SLP: lymphoproliferative syndrome, SMD: myelodysplastic syndrome, SMP: myeloproliferative syndrome, GIST: gastrointestinal stromal tumors*

^#^esophagus, stomach, duodenum, jejunum

^§^ileum, colon, rectum

**Table S3: Diagnostic performance of the TM panel for cancer**

|  | **CEA**  µg/L | **Total PSA**  ng/ml | **AFP**  ng/ml | **CA 125**  U/ml | **CA 15-3**  U/ml | **CA 19-9**  U/ml | **Calcitonin**  pg/ml | **NSE**  µg/L | **≥1 positive TMs** |
| --- | --- | --- | --- | --- | --- | --- | --- | --- | --- |
| Sensitivity [95%CI] | 0.38 [0.26-0.53] | 0.08 [0.03-0.20] | 0.12 [0.05-0.26] | 0.60 [0.44-0.74] | 0.31 [0.19-0.48] | 0.35 [0.22-0.50] | 0.05 [0.00-0.26] | 0.46 [0.29-0.65] | 0.80 [0.66-0.90] |
| Specificity [95%CI] | 0.86 [0.77-0.92] | 0.93 [0.85-0.97] | 0.97 [0.90-1.00] | 0.73 [0.60-0.83] | 0.89 [0.78-.095] | 0.95 [0.87-0.98] | 0.86 [0.71-0.94] | 0.81 [0.65-0.91] | 0.61 [0.50-0.71] |
| Prevalence [95%CI] | 0.37 [0.29-0.45] | 0.36 [0.28-0.45] | 0.36 [0.27-0.44] | 0.39 [0.29-0.48] | 0.38 [0.29-0.49] | 0.36 [0.27-0.44] | 0.36 [0.23-0.48] | 0.41 [0.29-0.53] | 0.37 [0.30-0.45] |
| PPV [95%CI] | 0.62 [0.44-0.80] | 0.40 [0.10-0.70] | 0.71 [0.38-1.00] | 0.58 [0.42-0.74] | 0.65 [0.42-0.87] | 0.79 [0.61-0.97] | 0.17 [0.00-0.47] | 0.63 [0.42-0.95] | 0.55 [0.43-0.66] |
| VPN [95%CI] | 0.70 [0.70-0.61] | 0.64 [0.55-0.73] | 0.67 [0.58-0.75] | 0.74 [0.63-0.85] | 0.68 [0.57-0.78] | 0.73 [0.64-0.81] | 0.62 [0.49-0.76] | 0.68 [0.54-0.82] | 0.84 [0.75-0.93] |
| LR+ [95%CI] | 2.79 [1.44-5.38] | 1.17 [0.35-3.93] | 4.52 [0.92-22.32] | 2.19 [1.34-3.60] | 2.93 [1.19-7.22] | 6.80 [2.41-19.21] | 0.36 [0.05-2.87] | 2.44 [1.11-5.35] | 2.06 [1.53-2.78] |
| LR- [95%CI] | 0.72 [0.56-0.91] | 0.99 [0.89-1.10] | 0.91 [0.81-1.02] | 0.56 [0.37-0.85] | 0.77 [0.60-0.98] | 0.69 [0.55-0.86] | 1.10 [0.94-1.30] | 0.66 [0.45-0.98] | 0.33 [0.18-0.58] |
| OR [95%CI] | 3.89 [1.66-9.13] | 1.18 [0.34-4.15] | 5.00 [1.07-23.46] | 3.94 [1.67-9.32] | 3.82 [1.30-11.20] | 9.91 [3.19-30.83] | 0.33 [0.05-2.16] | 3.67 [1.22-11.04] | 6.30 [2.82-14.10] |

*Legends: CI: confidence interval, LR +: positive likelihood ratio, LR -: negative likelihood ratio, NPV: negative predictive value, PPV: positive predictive value, OR: odds ratio.*
